# The association between Norton scale gain and functional outcome among older hip fracture patients

**DOI:** 10.1002/nop2.658

**Published:** 2020-10-17

**Authors:** Eliyahu H. Mizrahi, Emilia Lubart, Ilia Stambler, Abraham Adunsky

**Affiliations:** ^1^ Shmuel Harofe Geriatric Medical Center Beer Yaakov Israel; ^2^ Sackler School of Medicine Tel Aviv University Tel Aviv Israel; ^3^ Department of Geriatric Medicine and Rehabilitation Sheba Medical Center Tel‐Hashomer Israel

**Keywords:** elderly, functional outcome, hip fracture, Norton scale score, nurses, nursing, older people

## Abstract

**Aim:**

This study examines interrelations between gains of Norton Scale Score (NSS) and functional outcome measured by Functional Independence Measurement (FIM) among older hip fracture patients.

**Design:**

Retrospective study.

**Methods:**

We examined 227 patients consecutively hospitalized in a geriatric postacute rehabilitation ward. The data were collected during 2012‐2017. Data were analysed using Student's *t* test, chi‐square test, Pearson's correlation coefficient and linear regression.

**Results:**

Patients with positive NSS gains demonstrated statistically significant higher total FIM, motor FIM and total FIM gain scores at hospital discharge, compared with patients showing no NSS gains or negative NSS. Multiple regression analysis indicated that positive NSS gains were independently predictive for higher total FIM and motor FIM scores at hospital discharge and higher motor FIM gains at discharge.

**Conclusion:**

Our results suggest that positive NSS gains are associated with higher FIM scores at hospital discharge and may assist in predicting the functional outcome of hospitalized older hip fracture patients.

## 1 INTRODUCTION

Hip fractures commonly lead to hospitalization and disability of geriatric patients (Brainsky et al., 1997; Dyer et al., 2016; Gillespie, 2001). The functional outcomes for hip fracture patients depend on multiple aetiologic factors, including pre‐fracture function and health status, age, presence of anaemia, pain and type of the fracture (Kristensen, 2011; Meng et al., 2019). Various methods and scores are used to evaluate the patients function after hip fracture. These methods vary in their level of patient and personnel involvement, labour intensity, costs, predictive capabilities, thoroughness and accuracy (Reuben et al., 2017). It is clearly desirable to use evaluation methods that provide an adequate evaluation with good predictive capabilities, yet at the same time are inexpensive and labour‐saving. This study aims to contribute to the search for such efficient methods by testing and comparing the results of two evaluation methods commonly employed in functional assessment after hip fracture, namely the Functional Independence Measurement (FIM) scale scores and the Norton Scale Score (NSS).

## 2 BACKGROUND

The Functional Independence Measurement (FIM) scale is widely used to assess the level of disability of such patients and to monitor their progress throughout the rehabilitation process (Heinemann et al., 1993; Kristensen, 2011). The scale provides a grade for the functional status of a patient based on the level of assistance required, ranging from a total independence to total assistance. On the other hand, the Norton Scale Score (NSS) is a nursing‐based assessment tool that is generally used to predict the risk of suffering bedsores during the rehabilitation period (Norton et al., 1962) and consists of five domains. Despite various limitations of the NSS, the tool has been evaluated in aspects other than those relating to nursing.

Several authors (Gold et al., 2012; Justo et al., 2011) have demonstrated that low NSS at hospital admission is correlated with a higher incidence of postoperative complications, higher in‐hospital mortality, delayed rehabilitation and poor outcomes of rehabilitation in geriatric patients who underwent hip fracture surgery. However, none of these studies investigated the possible use of NSS gain results to predict FIM outcomes in this population. One major practical problem associated with the FIM use is the need for a comprehensive multidisciplinary staff (including a nurse, a physiotherapist, an occupational therapist and social worker) and a longer administration time. In contrast, the evaluation by NSS is simple, quick and can be easily performed by a single qualified nurse, thus reducing the burdens of the hospital staff using the FIM tool.

### 2.1 Research question

This study evaluates possible interrelations of FIM and NSS to determine whether NSS gain in older hip fracture patients is correlated with FIM scores at hospital discharge. Such data may assist in evaluating the degree of function of older hip fracture patients, based on NSS. In doing so, this study's purpose is to evaluate the NSS as a predictor of the FIM test in hopes of being able to substitute the complex FIM measurement. Insofar as the FIM evaluation generally requires an interdisciplinary rehabilitation team, the rationale for this research is to examine the possibility of substituting it with a simpler and quicker NSS test that can be performed by a nurse alone, in particular under conditions of shortage of multidisciplinary personnel. By comparing the results of these two common tests, this study aims to contribute to the recommendations for effective and at the same time economical functional evaluation tools.

## 3 THE STUDY

### 3.1 Design

This study is a retrospective chart survey based on the data of patients primarily diagnosed with hip fracture, who were admitted during 2012–2017 to geriatric postacute rehabilitation ward.

### 3.2 Method

#### 3.2.1 Setting

The routine rehabilitation process is a multifaceted intervention, performed by an interdisciplinary rehabilitation team. The team members convene two times a week to evaluate each patient's status and plan for the continuation of the rehabilitation treatment. A plan for the treatment is determined and monitored to coordinate and integrate the diverse treatment staff activities (including medical treatment, nursing, occupational and physical therapy and social work). The treated patients, as a rule, undergo physical and occupational therapy 6 hr a week. We used the STARD‐2015 checklist (STARD, 2015).

#### 3.2.2 Participants

The study comprised 227 geriatric patients consecutively admitted to a geriatric postacute rehabilitation ward from nearby orthopaedic departments, during the period 2012–2017. All patients suffered a traumatic low‐energy pertrochanteric (extra‐capsular) or sub‐capital (intra‐capsular) hip fracture, have undergone fracture fixation, were allowed full weight‐bearing and were in a stable medical condition enabling immediate active postoperative rehabilitation therapy.

We excluded patients with a rehabilitation period shorter than seven days (under the assumption that the degree of rehabilitation in such a short time is limited, possibly confounding the results). We also excluded patients with other severe disabilities, for example multiple traumas, medical conditions that would preclude active rehabilitation (e.g. severe chronic lung disease requiring constant oxygenation, cardiac failure in the functional capacity stage III‐IV of NYHC) and transition to acute care departments due to severe complications. Thus, patients were generally excluded who had either functional or medical conditions that may have impaired their rehabilitation capacity. Complete medical details were extracted from each patient's medical chart.

#### 3.2.3 Functional management

Patients were evaluated during hospital admission and hospital discharge for their functional ability, using the FIM and the NSS assessment tools. The FIM evaluation is commonly used to rate the patients' performance with five cognitive and 13 motor items.

The total FIM scores fall in the range between 18 (indicative of a total functional dependence)–126 (showing a total functional independence). In this study, the motor FIM was calculated separately, as it can be a highly sensitive measure to detect functional improvement in the patients.

The motor FIM measure is comprised of only 13 motor domains (i.e. excluding the five cognitive domains), and the scores fall in the range between 13 (the minimal score)–91 (the maximal score). In addition, we calculated the gains in total FIM and motor FIM scores, as the difference between the score at the hospital discharge and the score at the hospital admission.

Data about the patients' functional ability before the fracture were obtained from the patient or his/her family members and were classified as totally independent, partly and/or minimally dependent or totally dependent in activities of daily living and functional movement activities (Heinemann et al., 1993; Kristensen, 2011; Linacre, Heinemann, Wright, Granger, & Hamilton, 1994).

#### 3.2.4 Norton Scale Score

Norton Scale Score (NSS) is a simple scoring instrument that evaluates the general physical condition, level of activity, mental status, mobility and incontinence. The evaluation is performed by the nursing personnel at the hospital admission and discharge, with 1 (minimum) and 4 (maximum) points for each of its domains and with a final score falling in the range of 5 through 20 points (Norton et al., 1962).

#### 3.2.5 Cognitive management

The cognitive level of each patient was evaluated using the Mini‐Mental State Examination (MMSE) (Folstein et al., 1975).

### 3.3 Analysis

We compared patients showing positive (gain > 0) versus negative (gain ≤ 0) NSS gains (respectively, NSS‐PG and NSS‐NG) with reference to their clinical and functional measurements, using paired *t* tests for continuous variables and the chi‐square tests for dichotomous variables. We performed linear regression analysis between the patients' clinical, demographic, functional and cognitive characteristics and the total FIM or motor FIM scores at hospital discharge. The purpose of this analysis was to calculate the correlation between NSS and FIM, adjusted for the background, clinical and cognitive characteristics. The success of functional recovery at the end of the rehabilitation therapy was additionally estimated using the Montebello Relative Functional Scores (MRFS) (Drubach, Kelly, & Taragano, 1994).

The MRFS is calculated as discharge FIM minus admission FIM, divided by the maximum possible FIM minus admission FIM, the result multiplied by 100%. The results were considered statistically significant at *p* < .05. All statistical analyses were conducted with the SPSS SW for Windows, version 23, from IBM Inc. Power analysis was performed post hoc to validate the findings based on the sample *N* and effect‐size using the application G*Power, version 3.1.9.2.

### 3.4 Ethics

The study was approved by the Institutional Review Board of the Medical Center.

## 4 RESULTS

In examining the correlation between NSS and FIM assessments, we analysed the data on 227 geriatric hip fracture patients. The patients' mean age was 81.73 (*SD* 7.17 years old (mean ± *SD*). Most patients were females (62.1%). A positive gain in NSS at discharge was observed in 156 of the patients (68.7%).

First, we examined the associations between demographics and disease status on NSS changes. No statistically significant differences were seen between patients showing NSS positive gain (NSS‐PG, *N* = 156) and those with NSS negative gain (NSS‐NG, *N* = 71) with reference to gender, period of hospital stay and presence of major age‐related diseases in the inclusion criteria (including diabetes mellitus, hypertension, ischaemic heart disease, previous stroke, Parkinson's disease) and with reference to the pre‐fracture functional status. On the other hand, the patients' age was a significant determinant of the NSS changes.

The age of patients who showed a positive NSS gain was lower, and their MMSE score was higher compared with patients with NSS negative gain (*t* = 2.6, *df* = 225, *p* < .01; *t* = 2.9, *df* = 222, *p* < .01, respectively; Table 1).

**TABLE 1:** Clinical and cognitive characteristics of patients by Norton gain scores

Variable	All	PG	NPG	*p* *
Number	227	156	71	
Age, years	81.73 ± 7.17	80.9 ± 7.32	83.56 ± 6.5	.009
Female gender	141 (62.1)	93 (59.6)	48 (67.6)	.25
Length of stay, days	40.55 ± 13.93	41.22 ± 13.13	39.38 ± 15.44	.28
Diabetes mellitus	79 (34.8)	60 (38.5)	19 (26.8)	.086
Hypertension	171 (75.3)	118 (75.6)	513 (74.6)	.87
Ischaemic heart disease	124 (54.6)	89 (57.1)	35 (49.3)	.27
Previous stroke	32 (14.1)	18 (11.5)	14 (19.7)	.1
Parkinson's disease	16 (7)	12 (7.7)	4 (5.6)	.57
MMSE admission	17.85 ± 7.81	18.86 ± 7.27	15.63 ± 8.54	.004
Pre‐fracture level of independence, *N* (%)				
Independent		83 (53.2)	32 (45.1)	.49^**^
Partially dependent		53 (34)	27 (38)	
Totally dependent		20 (12.8)	12 (16.9)	

Abbreviation: PG ‐ positive gain (*x* > 0), NPG ‐ no positive gain (*x* ≤ 0).

* *p* calculated using chi‐squared test for categorical variables and Student's *t* test for comparisons of continuous variables.

^**^ *p*‐value refers to the association between Norton gain and pre‐fracture level of Independence.

The bivariate associations between NSS and FIM were significant both at admission (*r* = 0.71, *p* < .001) and discharge (*r* = 0.76, *p* < .001). Comparing NSS and FIM changes, we found no statistically significant differences between NSS‐PG and NSS‐NG patient groups with reference to their total FIM (*t* = 0.13, *df* = 225, *p* = .89) and motor FIM scores (*t* = 1.21, *df* = 225, *p* = .22) at hospital admission (Table 2). However, patients demonstrating positive NSS gain had a significantly higher total FIM (*t* = 4.97, *df* = 225, *p* < .001) and motor FIM scores (*t* = 5.42, *df* = 225, *p* < .001) at hospital discharge. Also, the FIM gain scores for both the total FIM (*t* = 8.48, *df* = 225, *p* < .001) and motor FIM (*t* = 9.56, *df* = 225, *p* < .001) were higher in the NSS‐PG group as compared with the NSS‐NG group (Table 2).

**TABLE 2:** Functional characteristics of patients by Norton gain scores at admission and discharge

Variable	PG	NPG	*p* *
Admission total FIM	63.01 ± 15.24	63.28 ± 15.24	.894
Discharge total FIM	86.1 ± 14.74	74.72 ± 18.45	<.001
Gain in total FIM	23.09 ± 9.43	11.43 ± 9.97	<.001
Admission motor FIM	38.08 ± 8.77	39.69 ± 10.12	.224
Discharge motor FIM	58.87 ± 11.23	49.62 ± 13.26	<.001
Gain in motor FIM	20.78 ± 7.58	9.92 ± 8.65	<.001
MFRS	0.375 ± 0.147	0.196 ± 0.179	<.001

Abbreviations: MFRS, Montebello Relative Functional Scores; NPG, No positive gain; PG, positive gain.

* *p* calculated using Student's *t* test for comparisons of continuous variables.

**FIGURE 1:**
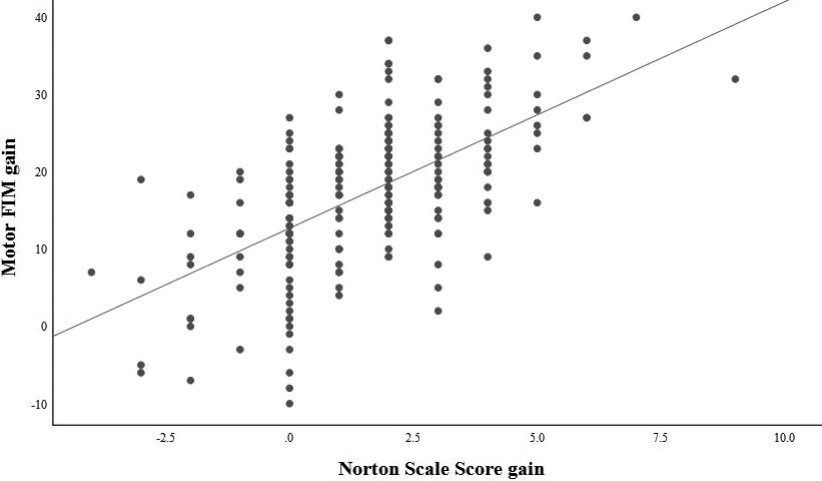
Correlation of motor Functional Independence Measurement (FIM) gain with Norton Gain at discharge (Spearman correlation coefficient beta = 0.611, *p* < .001)

The NSS‐PG patients also showed higher Montebello Relative Functional Scores (MRFS) compared with NSS‐NG patients when controlling for a possible “ceiling effect” (0.38 *SD* 0.15, 0.20 *SD* 0.18, *p* < .001, respectively). Figure 1 shows the correlation of motor Functional Independence Measurement gain with Norton Score gain at discharge. As shown in Figure 1, there was a statistically significant correlation between positive NSS gain and motor FIM gain at hospital discharge (*r*_s_ = 0.61, *p* < .001). Thus, it is evident that NSS gains are generally positively correlated with functional status and improvements as shown by FIM measurements.

Next, we aimed to minimize potential confounding effects by finding independent predictors. Since the NSS‐PG patients' group was slightly younger, a linear regression model was used to find possible independent predictors of functional outcome at hospital discharge.

According to our results (shown in Table 3), the total FIM, motor FIM and motor FIM gain (at hospital discharge) were independently and inversely correlated with the patients' age (beta = −0.133, *p* = .003; beta = −0.137, *p* = .003; beta = −0.117, *p* = .03, respectively) and with the pre‐fracture functional level (beta = −0.305, *p* < .001; beta = −0.325, *p* < .001; beta = −0.139, *p* = .017, respectively).

**TABLE 3:** Multiple regression analysis of factors predicting FIM scores at discharge

Variable	Total FIM*		Motor FIM*		Motor FIM gain*	
	*β*	*p*	*β*	*p*	*β*	*p*
Norton gain scores	0.235	<.001	0.271	<.001	0.534	<.001
Age (years)	−0.133	.003	−0.137	.003	−0.117	.03
Gender (female)	0.1	.017	0.074	.087	0.05	.329
Length of stay (days)	0.047	.255	0.027	.532	0.092	.068
Ischaemic heart disease	−0.073	.134	−0.066	.19	−0.016	.792
Arterial hypertension	0.012	.794	−0.012	.785	−0.037	.491
Diabetes mellitus	−0.02	.677	−0.033	.509	0.047	.424
Parkinson's disease	−0.095	.02	−0.098	.021	−0.053	.287
Previous stroke	−0.025	.541	−0.054	.208	−0.058	.252
MMSE	0.466	<.001	0.41	<.001	0.185	.002
Pre‐fracture level of function	−0.305	<.001	−0.325	<.001	−0.139	.017

* *R* ^2^ = .667; ^**^ *R* ^2^ = .648; ^†^ *R* ^2^ = .509.

Moreover, a higher MMSE score at hospital admission emerged as a significant factor predictive independently for higher total FIM, motor FIM and FIM gain scores at hospital discharge (beta = 0.466, *p* < .001; beta = 0.41, *p* < .001; beta = 0.185, *p* = .002, respectively). No other tested variable was predictive for higher total FIM or motor FIM scores at hospital discharge. Generally, we obtained satisfactory *R* ^2^ values of .667 and .648, respectively, for total FIM and motor FIM at hospital discharge and *R* ^2^ value of .509 for motor FIM gain at hospital discharge. The results indicate that these variables account for most of the total FIM score variance.

## 5 DISCUSSION

The present results show that older hip fracture patients who exhibit positive Norton Scale Scores (NSS) gains also demonstrate higher scores for total FIM, motor FIM and motor FIM gain on discharge, compared with patients that showed either no gain or a negative NSS gain. In addition, the linear regression analysis adjusting for multiple confounders, such as age, gender, length of hospital stay and presence of multimorbidity showed that positive NSS gain may serve as a statistically significant independent predictor for total FIM, motor FIM and motor FIM gain at hospital discharge. Furthermore, the bivariate associations between NSS and FIM were significant both at admission and discharge. This strengthens the assumption that there exists a strong correlation between these two variables, thus further supporting their potential interchangeability. Importantly, the power analysis of the above findings indicated very high 1−*β* values, approaching *p* = 1.0.

These results are in accordance with and supportive of the results of previous studies (Gold et al., 2012; Halperin et al., 2012) that investigated factors related to prognosis for patients hospitalized in orthopaedic and rehabilitation departments and underscore the prognostic significance of NSS in older hip fracture patients following surgery.

The present study adds to the subject field, compared with previous data, with the following aspects. First, one of the above mentioned studies (Halperin et al., 2012) used only walking FIM scores to evaluate the outcome, thus referred solely to the walking ability at discharge, rather than the overall functional outcome in our study using data for all FIM items.

Second, an earlier study (Gold et al., 2012) reporting low Norton Scale Scores at admission did not associate with functional outcomes and used only the Norton data without using FIM or other commonly used functional outcome scores. Finally, in the present study, the results were also verified for a possible ceiling effect as manifested by the significantly higher Montebello Relative Functional Scores (MRFS).

These results are in line with the hypothesis that NSS might be used for prognostication of older hip fracture patients, as this score comprises several basic health aspects of the older population (Norton et al., 1962). Therefore, the use of the simpler and less labour‐consuming NSS may help overcome the increasing geriatric care deficits associated with the chronic shortage of professional workers in the field of geriatric care services (Bo et al., 2008; Landry et al., 2007).

These deficits are particularly strongly felt in suburban areas, even in the developed countries, where such professional workforce shortages are common (Bo et al., 2008; Landry et al., 2007; O'Toole & Schoo, 2010; Stanmore & Waterman, 2007; Wilson et al., 2009). This application may be relevant not only for geriatric rehabilitation processes being performed during hospitalization, but also for community‐delivered rehabilitation programmes that constitute an important element of the older care sequence (Turner‐Stokes et al., 2006). Such programmes are also subjected to geriatric workforce shortages under global changing demographics (World Health Organization, 2010). In terms of convenience, accessibility, cost and labour‐savings, NSS may be advantageous over FIM evaluation.

Unlike the NSS, the administration of FIM scores is not easy or quick and requires a significant deal of expertise, training and experience in the scoring system. It is also time‐consuming as it takes approximately 20–30 min to complete and involves evaluation by multidisciplinary professional staff.

Being much simpler, shorter, more reliable and feasible, the NSS may replace the FIM as a prognostic outcome predictor for this population. Presumably, FIM score may be a more sensitive tool to identify changes of function, compared with NSS. However, the documentation of rehabilitation processes by NSS is outside the scope of this study that primarily aimed to investigate whether NSS may assist in predicting functional outcome in this population.

### 5.1 Limitations

Our study may have several limitations resulting from its retrospective nature, being performed in a single medical centre and comprising a relatively small number of enrolled patients. The study also did not evaluate or compare the reliability of NSS versus the FIM, but only investigated the interrelations of NSS with functional outcomes as estimated by the FIM. Under the present limited sample size, the only way to show the potential interchangeability of these two tests was to show the correlation between them, as was done in the present study. However, in future studies, with more data, it is hoped that it will be possible to validate the reproducibility of predictions by these two tests. Despite these limitations, this research may be beneficial for medical wards dedicated to treating such patients where all patients undergo a standard rehabilitation programme.

## 6 CONCLUSIONS

We conclude that positive gains of Norton Scale Score (NSS‐PG) are correlated with better functional outcomes in older hip fracture patients (as evaluated by FIM). Following additional validation, these findings may assist health policy decision‐makers involved in treatment management and financing of such patients. Based on the present limited sample size, it is not possible to provide definite clinical recommendations. However, the present study does indicate the possibility of substituting NSS for the FIM as an outcome predictor. Clearly, this requires further research with more extensive data.

Dependent on the further validation of this possibility and insofar as NSS assessment is simpler, shorter, more reliable and feasible, the NSS evaluation by the nursing staff may replace the FIM as a routine prognostic outcome predictor for older hip fracture patients, as it would minimize the need for multidisciplinary professional staff. Therefore, the use of NSS may help overcome the increasing care deficit associated with the chronic shortage of professional geriatric care workers. Thus, the nursing personnel may provide service and solutions in areas beyond its original scope of activity.

## CONFLICT OF INTEREST

The authors have no conflict of interest.

## AUTHOR CONTRIBUTIONS

EHM, AA designed the study. EHM, EL collected and analysed the data. EHM, IS and AA prepared the manuscript.

## Data Availability

The anonymized patient data are available at the Shmuel Harofe Geriatic Medical Center, Israel, subject to IRB approval.
